# Dietary Antibiotic Growth Promoters Down-Regulate Intestinal Inflammatory Cytokine Expression in Chickens Challenged With LPS or Co-infected With *Eimeria maxima* and *Clostridium perfringens*

**DOI:** 10.3389/fvets.2019.00420

**Published:** 2019-11-22

**Authors:** Sungtaek Oh, Hyun S. Lillehoj, Youngsub Lee, David Bravo, Erik P. Lillehoj

**Affiliations:** ^1^Animal Bioscience and Biotechnology Laboratory, Beltsville Agricultural Research Center, Agricultural Research Service, United States Department of Agriculture, Beltsville, MD, United States; ^2^Pancosma, Geneva, Switzerland; ^3^Department of Pediatrics, University of Maryland School of Medicine, Baltimore, MD, United States

**Keywords:** poultry production, cytokine, inflammation, antibiotics, necrotic enteritis, *Eimeria*

## Abstract

Subtherapeutic levels of dietary antibiotics increase growth performance in domestic animals, but the mechanisms are poorly understood. Here, 1-week-old broiler chickens were challenged with LPS (experiment 1), or co-infected with *Eimeria maxima* and *Clostridium perfringens* as an experimental model of necrotic enteritis (experiment 2), and fed a standard basal diet or a basal diet supplemented with virginiamycin or bacitracin methylene disalicylate. In experiment 1, LPS-challenged chickens fed the unsupplemented diet had decreased body weight gains, compared with unsupplemented controls given the PBS control. In contrast, antibiotic supplementation increased body weight gains in both the LPS-challenged and PBS groups, compared with the antibiotic-free diet. LPS-challenged chickens fed the unsupplemented diet had increased expression levels of intestinal tight junction proteins (ZO1, JAM2), MUC2 gel-forming mucin, and inflammatory cytokines (IL-1β, IL-2, IL-6, IL-8, IL-17A) at 24 h post-challenge, compared with unsupplemented chickens given the PBS control. However, LPS-challenged chickens fed the antibiotic-supplemented diets had decreased levels of intestinal inflammatory cytokine transcripts, compared with LPS-challenged chickens given the unsupplemented basal diet. In experiment 2, *E. maxima*/*C. perfringens*-co-infected chickens fed the antibiotic-supplemented diets had increased body weight gains, decreased intestinal pathology, and greater intestinal crypt depth, compared with co-infected chickens given the unsupplemented diet. Further, similar to LPS challenge, *E. maxima*/*C. perfringens*-co-infection of chickens fed the antibiotic-supplemented diets decreased expression levels of intestinal inflammatory cytokines, compared with co-infected chickens given the unsupplemented diet. These results support the hypothesis that dietary antibiotic growth promoters might increase poultry growth, in part, through down-regulation of pathogen-induced inflammatory responses.

## Introduction

The first report of antibiotics as animal growth promoters was published in 1946 (1). In the subsequent 70 years, antibiotic growth promoters (AGPs) have been commercialized as non-therapeutic dietary supplements to improv food animal growth and feed conversion efficiency, prevent infectious diseases, and maximize economic profits (2). However, more recent efforts have focused on reducing or eliminating AGP use with the realization that overuse of AGPs in agricultural animals leads to the development of antibiotic resistant bacteria with the potential to spread to the human population (3, 4). Antibiotic-resistant bacteria are estimated to kill an extra 10 million people annually worldwide by 2050 (5). As a result, the U.S. Food and Drug Administration has requested that pharmaceutical companies voluntarily discontinue labeling antimicrobials for growth promotion in agricultural animals, and antibiotics should only be prescribed for therapeutic uses through veterinary oversight (6). Among the new initiatives to eliminate AGP use in food animals is the development of alternatives to antibiotics that improve growth performance while maintaining optimal animal health. Some of the alternatives that have been described are probiotics, prebiotics, synbiotics, organic acids, enzymes, and phytochemicals (7).

A comprehensive understanding of the biological mechanism of action of AGPs is needed in order to develop effective non-antibiotic alternatives that retain their growth enhancing properties without promoting antimicrobial resistance. However, limited information exists on the cellular and molecular processes through which AGPs improve animal growth, although several hypotheses have been proposed (8–11). One theory posits that consumption of AGPs reduces the quantity and diminishes the diversity of the gut microbiota, particularly for Gram-positive microorganisms, which interfere with the intestinal absorption of nutrients essential for growth (8, 9). An alternative suggestion proposes that following an inflammatory stimulus, such as exposure to infectious pathogens and their toxins, stress, or trauma, AGPs impart an anti-inflammatory effect by reducing the production of cytokines and chemokines, leading to decreased muscle catabolism and diminished anorexia (10). More specifically, Niewold (10) proposed that AGPs suppress the production of catabolic mediators by intestinal inflammatory cells, thereby altering the normal microflora, while others suggest a reverse relationship as being most dominant, i.e., AGPs induce microbiota changes that reduce inflammatory interactions with the intestine (12–20). In general, inflammatory responses are complex, protective biological responses to pathogens, damaged cells, or irritants. We previously reported that virginiamycin and bacitracin methylene disalicylate, two commonly-used AGPs in commercial poultry production, alter the intestinal metabolome in naïve chickens (21). The current study was undertaken to determine whether these two AGPs also might alter chicken intestinal physiology and/or gut inflammation in response to challenge by bacterial lipopolysaccharide (LPS) or co-infection with *Eimeria maxima* and *Clostridium perfringens* as an experimental model of avian necrotic enteritis.

## Materials and Methods

### Experimental Design

#### Overall Design

**Figure 1:**
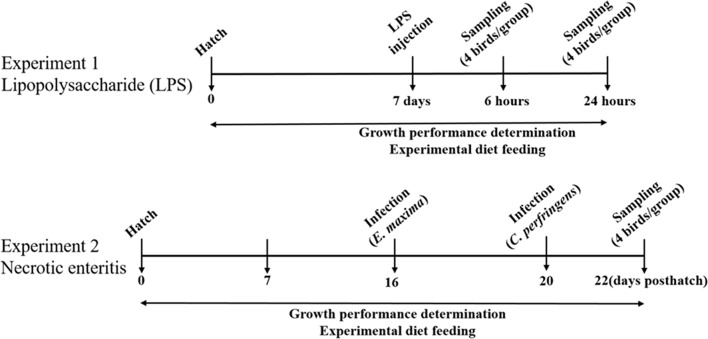
Schematic outline of the experimental design.

The overall objectives of this study are to characterize the physiological and inflammatory responses of chickens fed an AGP-supplemented diet compared with those receiving an unsupplemented diet. To achieve these objectives, we measured the levels of gene transcripts encoding for intercellular tight junction proteins, proinflammatory cytokines, and antioxidant enzymes in different tissues (intestinal jejunum vs. liver), and following different challenges (LPS injection vs. *E. maxima*/*C. perfringens* co-infection), different routes of administration (intraperitoneal vs. oral gavage), different chicken ages (7 days vs. 16–22 days), and different sampling times post-challenge (hours vs. days) (Figure 1).

#### Experiment 1

All experiments were approved by the Beltsville Agricultural Research Center Institutional Animal Care and Use Committee. Two hundred and forty, 1-day-old Ross 708 male broiler chickens were obtained from Longenecker's Hatchery (Elizbethtown, PA), housed in Petersime starter brooder units, and provided with feed and water *ad libitum*. The experimental design consisted of a randomized 2 × 3 factorial arrangement with LPS- or PBS vehicle-administered chickens fed a basal diet, or a diet supplemented with virginiamycin or bacitracin methylene disalicylate (Figure 1). Chickens (40/group, 8/cage) were fed from hatch with a corn- and soybean meal-based unsupplemented, basal diet containing 24% dry matter protein formulated to meet or exceed the National Research Council's 1994 nutrient requirements for broiler chickens (22), or the basal diet supplemented with 20 g/ton (22 ppm) of virginiamycin (Phibro Animal Health, Teaneck, NJ) or 50 g/ton (55 ppm) of bacitracin methylene disalicylate (Zoetis, Durham, NC) (21). At 7 days of age, chickens were injected intraperitoneally with 1.0 mg/kg body weight of *Escherichia coli* O55:B5 LPS (Sigma, St. Louis, MO) or with sterile PBS.

#### Experiment 2

All experiments were approved by the Beltsville Agricultural Research Center Institutional Animal Care and Use Committee. One hundred and forty 1-day-old Ross 708 male broiler chickens were randomly divided into 4 groups (35 chickens/group, 7 chickens/cage): (1) uninfected with basal diet alone, (2) *E. maxima*/*C. perfringens* co-infected with basal diet alone, (3) *E. maxima*/*C. perfringens* co-infected with basal diet containing virginiamycin (20 g/ton, 22 ppm), or (4) *E. maxima*/*C. perfringens* co-infected with basal diet containing bacitracin methylene disalicylate (50 g/ton, 55 ppm) (Figure 1). The basal diet contained 18% (w/w) dry matter protein (USDA-Feed Mill, Beltsville, MD) between days 1 and 20 post-hatch and a standard grower diet containing 24% (w/w) dry matter protein between days 20 and 28, as described (23). At day 16 post-hatch, chickens were infected by oral gavage with 1.0 × 10^4^ oocysts/bird of *E. maxima* Beltsville strain 41A. At day 20, the chickens were infected by oral gavage with 1.0 × 10^9^ colony forming units/bird of *C. perfringens* strain Del-1. Uninfected birds received an equal volume of PBS by oral gavage. Uninfected chickens were housed in same room as infected birds, but in different room partitions and different cages to prevent cross-infection.

### Body Weights and Tissue Collection

For experiment 1, body weights were recorded daily, and at 6 and 24 h following LPS challenge (Figure 1). At 6 and 24 h post-challenge, chickens (4/group) were randomly selected and euthanized by cervical dislocation. The bursa of Fabricius and spleen were immediately removed, blotted dry, weighed, and organ weight calculated relative to 100 g of body weight. Intestinal jejunum tissues were collected and immediately stored at −20°C in RNAlater stabilization solution (Applied Biosystems, Foster City, CA) for RNA extraction. Intestinal duodenum, jejunum, and ileum tissues were fixed in 10% neutral buffered formalin, embedded in paraffin, and 5.0 μm tissue sections were prepared, stained with haematoxylin and eosin, and examined at 100X using an Eclipse T-E inverted microscope (Nikon, Melville, NY). Intestinal crypt depth and villus height were measured as described (24). Four microscopic fields per sample were measured. For experiment 2, body weights were recorded daily. Intestinal jejunum and liver tissues (4/group) were collected at days 22 and 28 post-hatch and stored at −20°C in RNAlater solution. Intestinal lesion scores (jejunum) were measured at day 22 post-hatch on a scale from 0 (none) to 4 (high) by 4 independent observers in a blinded fashion as described (24). The intestinal jejunum was selected for analysis because this region contains the highest numbers of parasite developmental stages following *E. maxima* infection (25).

### RNA Isolation and qRT-PCR

Intestinal jejunum and liver samples (4/group) were homogenized individually using a hand-held rotor-stator homogenizer (TissueRuptor, Qiagen, Germantown, MD). Total RNA was extracted using TRIzol (Invitrogen, Carlsbad, CA), purified with the RNeasy Mini RNA purification kit (Qiagen) and 5.0 μg of total RNA were treated with 1.0 U of DNase I (Sigma) for 15 min at 22°C followed by 10 min at 70°C. The RNA was reverse-transcribed using the StrataScript first-strand synthesis system (Stratagene, La Jolla, CA) according to the manufacturer's recommendations. cDNA was amplified using the Mx3000P QPCR system (Agilent Technologies, Santa Clara, CA) and Brilliant SYBR Green qPCR master mix (Stratagene) for 40 cycles at 72°C for 1 min with oligonucleotide primers for chicken interleukin (IL)-1β, IL-2, IL-6, IL-8, IL-17A, lipopolysaccharide-induced tumor necrosis factor-α-factor (LITAF), catalase (CAT), heme oxygenase 1 (HMOX1), superoxide dismutase 1 (SOD1), zona occludens-1 (ZO1), junctional adhesion molecule-2 (JAM2), and mucin-2 (MUC2) (Table 1). The levels of individual transcripts were normalized to those of GAPDH by the Q-gene program (24). To normalize individual replicates, logarithmic-scaled threshold cycle (C_t_) values were transformed to linear units of normalized expression before calculating the mean and standard errors of the mean (SEM) for the reference and target transcripts, followed by determination of mean normalized expression.

**Table 1:** Primers used for qRT-PCR.

**RNA target**	**Primer sequence**	**GenBank accession no**.
GAPDH	F: 5′-GGTGGTGCTAAGCGTGTTAT-3′	NM_204305
	R: 5′-ACCTCTGTCATCTCTCCACA-3′	
IL-1β	F: 5′-TGGGCATCAAGGGCTACA-3′	NM_204524
	R: 5′-TCGGGTTGGTTGGTGATG-3′	
IL-2	F: 5′-TCTGGGACCACTGTATGCTCT-3′	NM_204153.1
	R: 5′-ACACCAGTGGGAAACAGTATCA-3′	
IL-6	F: 5′-CAAGGTGACGGAGGAGGAC-3′	NM_204628
	R: 5′-TGGCGAGGAGGGATTTCT-3′	
IL-8	F: 5′-GGCTTGCTAGGGGAAATGA-3′	NM_205498.1
	F: 5′-AGCTGACTCTGACTAGGAAACTGT-3′	
IL-17A	F: 5′-CTCCGATCCCTTATTCTCCTC-3′	NM_204460.1
	R: 5′-AAGCGGTTGTGGTCCTCAT-3′	
LITAF	F: 5′-TGTGTATGTGCAGCAACCCGTAGT-3′	NM_204267
	R: 5′-GGCATTGCAATTTGGACAGAAGT-3′	
CAT	F: 5′-ACTGCAAGGCGAAAGTGTTT-3′	NM001031215.1
	R: 5′-GGCTATGGATGAAGGATGGA-3′	
HMOX1	F: 5′-CTGGAGAAGGGTTGGCTTTCT-3′	NM205344
	R: 5′-GAAGCTCTGCCTTTGGCTGTA-3′	
SOD1	F: 5′-ATTACCGGCTTGTCTGATGG-3′	NM205064.1
	R: 5′-CCTCCCTTTGCAGTCACATT-3′	
ZO1	F: 5′-CCGCAGTCGTTCACGATCT-3′	XM_015278981.1
	R: 5′-GGAGAATGTCTGGAATGGTCTGA-3′	
JAM2	F: 5′-AGCCTCAAATGGGATTGGATT-3′	NM_001006257.1
	R: 5′-CATCAACTTGCATTCGCTTCA-3′	
MUC2	F: 5′-GCCTGCCCAGGAAATCAAG-3′	NM_001318434.1
	R: 5′-CGACAAGTTTGCTGGCACAT-3′	

### Statistical Analysis

All data were expressed as mean ± SEM values for each treatment group. Tests of normality were performed on all the data. Mean values between treatment groups were compared by two-way ANOVA with Duncan's multiple range test using SAS software (version 9.4, SAS Institute, Cary, NC). Main factor and interaction of main factor comparisons were made between AGP dietary supplementation and LPS challenge. One-way ANOVA with Duncan's multiple range test was used to compare unsupplemented vs. AGP-supplemented diets in *E. maxima*/*C. perfringens* co-infected chickens, as described (24). *P* < 0.05 were considered significantly different.

## Results

### Experiment 1

#### Growth Performance and Lymphoid Organ Weights

LPS-challenged chickens fed the unsupplemented diet had decreased body weight gains at both 6 and 24 h post-challenge, compared with unsupplemented chickens given the PBS vehicle alone (Table 2). A similar result was seen in LPS-administered chickens fed a diet containing the AGPs, virginiamycin or bacitracin methylene disalicylate, compared with the PBS controls. In contrast, when comparing body weight gains in the AGP-supplemented group between PBS and LPS, AGP supplementation increased weight gains both prior to and at 6 and 24 h following PBS or LPS administration, compared with the antibiotic-free diet. The only observed effect of LPS administration or AGP dietary supplementation on normalized weights of the bursa of Fabricius or spleen was an increase in spleen weight in LPS-challenged chickens fed the AGP-supplemented diet at 6 h post-challenge, compared with the PBS controls (Table 3).

**Table 2:** Body weights of chickens fed an unsupplemented diet or a diet supplemented with virginiamycin or bacitracin methylene disalicylate with or without LPS.

		**Body weight (g/chicken)**							
**Days post-hatch**		**1**	**2**	**3**	**4**	**5**	**6**	**7**		
**Hours post-injection**								**0**	**6**	**24**
PBS	Basal diet	53.6	62.5	79.2	96.7	118.7	139.4	162.3	171.1	188.7
	Virginiamycin	53.8	63.8	79.1	99.8	121.3	137.5	159.1	168.9	191.4
	BMD^a^	54.5	66.0	80.7	100.0	122.8	142.0	164.7	174.6	196.4
LPS	Basal Diet	54.0	64.2	78.3	95.5	117.2	136.9	159.0	156.0	174.1
	Virginiamycin	53.6	63.4	78.6	97.5	120.0	139.5	163.3	159.3	177.2
	BMD	54.7	65.5	81.7	100.9	124.1	145.9	170.1	166.4	185.0
Pooled SEM	Treatments	0.54	0.75	1.20	1.62	2.15	2.61	3.18	3.54	4.38
	PBS vs. LPS	0.31	0.43	0.69	0.94	1.24	1.51	1.84	2.05	2.53
	Basal vs. AGP	0.38	0.53	0.85	1.15	1.52	1.85	2.25	2.50	3.10
Pooled *P*-value	Treatments	0.600	0.013	0.305	0.124	0.216	0.163	0.141	0.002	0.003
	PBS vs. LPS	0.833	0.660	0.900	0.504	0.767	0.604	0.422	0.0002	0.0002
	Basal vs. AGP	0.196	0.003	0.070	0.027	0.040	0.048	0.064	0.097	0.100
	LPS × AGP^b^	0.848	0.275	0.717	0.627	0.775	0.468	0.342	0.594	0.923

a *Bacitracin methylene disalicylate*.

b *Interaction between AGP supplementation and LPS administration*.

**Table 3:** Bursa of Fabricius and spleen weights of chickens fed an unsupplemented diet or a diet supplemented with virginiamycin or bacitracin methylene disalicylate with or without LPS^a^.

		**Organ weight (g/chicken, normalized to 100 g body weight)**					
		**Bursa of fabricius**			**Spleen**		
**Hours post-injection**		**0**	**6**	**24**	**0**	**6**	**24**
PBS	Basal diet	0.213	0.151	0.173	0.095	0.090	0.100
	Virginiamycin	0.185	0.192	0.186	0.112	0.095	0.083
	BMD^a^	0.183	0.184	0.192	0.100	0.110	0.118
LPS	Basal Diet	0.181	0.212	0.162	0.096	0.141	0.112
	Virginiamycin	0.217	0.166	0.191	0.112	0.142	0.115
	BMD	0.162	0.199	0.142	0.102	0.128	0.112
SEM	Untreated vs. Treated	0.024	0.020	0.019	0.010	0.014	0.013
	PBS vs. LPS	0.014	0.011	0.011	0.006	0.008	0.007
	Basal vs. AGP	0.017	0.014	0.013	0.007	0.011	0.009
Pooled *P*-value	Untreated vs. Treated	0.588	0.320	0.431	0.747	0.080	0.407
	PBS vs. LPS	0.738	0.318	0.253	0.946	0.005	0.235
	Basal vs. AGP	0.449	0.796	0.462	0.290	0.971	0.453
	LPS × AGP^b^	0.383	0.118	0.365	0.994	0.463	0.354

a *Bacitracin methylene disalicylate*.

b *Interaction between AGP supplementation and LPS administration*.

#### ZO1, JAM2, and MUC2 Transcript Levels

**Figure 2:**
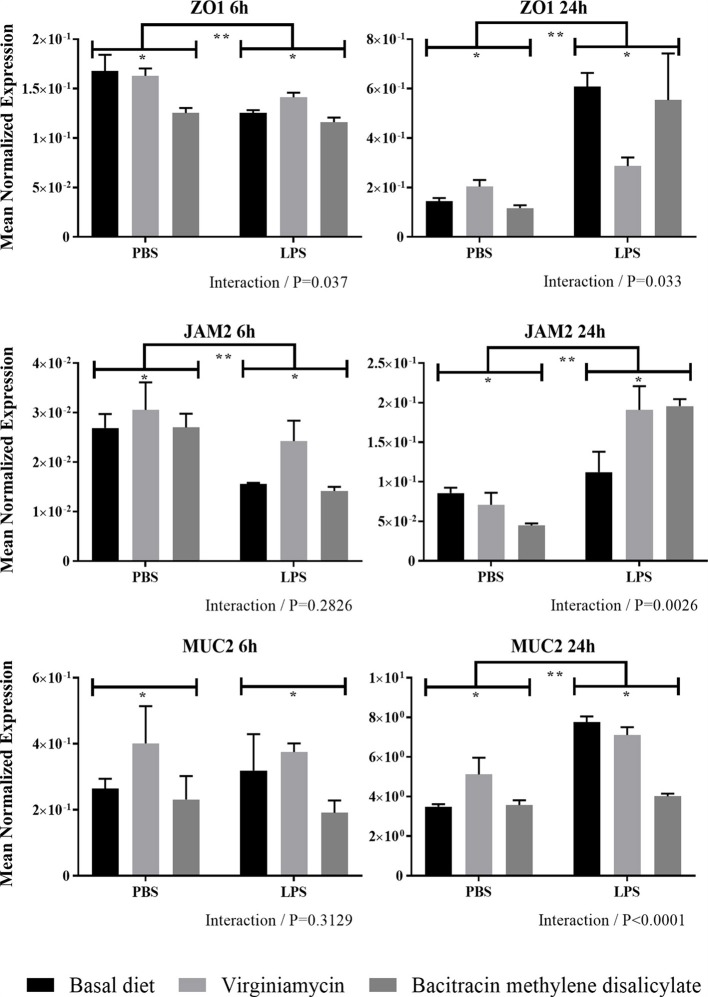
Transcript levels of ZO1, JAM2, and MUC2 expression in intestine with or without LPS challenge. Chickens were fed from hatch with an unsupplemented basal diet or a diet supplemented with 20 g/ton of virginiamycin or 50 g/ton of bacitracin methylene disalicylate. At 7 days post-hatch, the chickens were injected intraperitoneally with 1.0 mg/kg body weight of *E. coli* O55:B5 LPS or PBS. At 6 and 24 h post-injection, the levels of intestinal jejunum transcripts encoding ZO1, JAM2, and MUC2 were measured by qRT-PCR and normalized to GAPDH transcripts. Vertical bars represent mean ± SEM normalized transcript levels (*n* = 4). *Significant difference between chickens fed an AGP-supplemented diet compared with unsupplemented controls at *P* < 0.05. **Significant difference between LPS-injected chickens compared with PBS controls at *P* < 0.05. *P*-values for the interaction between AGP supplementation and LPS administration are indicated below each panel.

In general, LPS-challenged chickens fed the unsupplemented or AGP-supplemented diet had increased levels of intestinal transcripts encoding ZO1 and JAM2 at 24 h compared with 6 h (Figure 2). LPS-challenged chickens fed the unsupplemented diet or an AGP-supplemented diet had decreased levels of intestinal transcripts encoding ZO1 and JAM2 at 6 h post-challenge, but increased transcript levels at 24 h, compared with unsupplemented chickens given the PBS vehicle alone (Figure 2). In contrast, no consistent changes in the levels of either transcript were observed when comparing the unsupplemented vs. AGP-supplemented diets in either the PBS- or LPS-administered chickens at 6 or 24 h post-administration. Significant interactions between AGP supplementation and LPS challenge on altered ZO1 transcript levels were observed at 6 and 24 h post-administration, and for JAM2 transcript levels at 24 h. At this later time point, intestinal MUC2 transcript levels were increased in LPS-challenged chickens fed both the unsupplemented or AGP-supplemented diets, compared with the PBS controls, and a significant interaction between AGP supplementation and LPS administration was observed (Figure 2).

#### Proinflammatory Cytokine and Antioxidant Enzyme Transcript Levels

**Figure 3:**
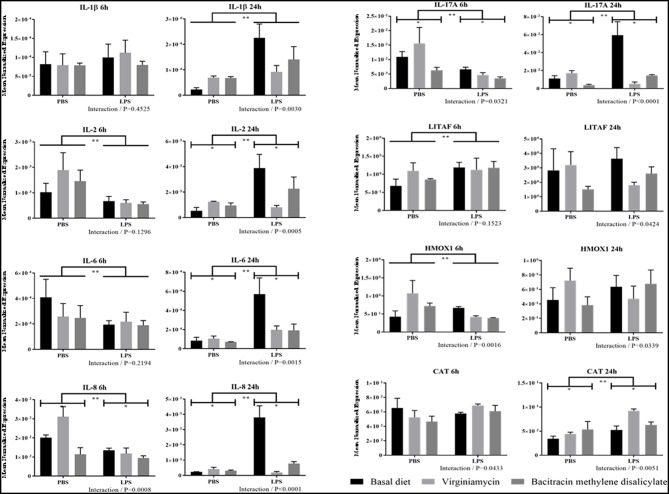
Transcript levels of proinflammatory cytokine and antioxidant enzyme expression in intestine with or without LPS challenge. Chickens were fed an unsupplemented basal diet or a diet supplemented with virginiamycin or bacitracin methylene disalicylate, challenged with LPS or PBS, and the levels of intestinal transcripts encoding IL-1β, IL-2, IL-6, IL-8, IL-17A, LITAF, HMOX1, and CAT were measured by qRT-PCR and normalized to GAPDH transcripts. Vertical bars represent mean ± SEM normalized transcript levels (*n* = 4). *Significant difference between chickens fed an AGP-supplemented diet compared with unsupplemented controls at *P* < 0.05. **Significant difference between LPS-injected chickens compared with PBS controls at *P* < 0.05. *P*-values for the interaction between AGP supplementation and LPS administration are indicated below each panel.

With the exception of LITAF transcripts, LPS-challenged chickens fed the unsupplemented basal diet had consistently increased levels of intestinal transcripts encoding IL-1β, IL-2, IL-6, IL-8, and IL-17A at 24 h post-challenge, compared with unsupplemented chickens given PBS alone (Figure 3). No consistent alterations in transcript levels were evident between these two treatment groups at 6 h post-challenge. In contrast, AGP-supplementation in LPS-administered chickens was consistently associated with reduced levels of all proinflammatory cytokine transcripts examined at 24 h post-administration, compared with LPS-challenged chickens fed the unsupplemented diet. Again, no differences in cytokine transcript levels were seen at 6 h post-challenge. No consistent changes in these transcripts were evident at either 6 or 24 h when comparing LPS-challenged chickens fed the AGP-supplemented diet vs. PBS controls, or between chicken given the AGP-supplemented vs. unsupplemented diets in the absence of LPS administration. Significant interactions between AGP supplementation and LPS administration on altered transcript levels were observed at 6 h for IL-8 and IL-17A, and at 24 h for all cytokines examined. The levels of transcripts encoding for CAT or HMOX1 were, for the most part, unchanged in PBS- vs. LPS-administered chickens given the unsupplemented or AGP-supplemented diets, and in chickens fed unsupplemented vs. AGP-supplemented diets and administered PBS or LPS (Figure 3). However, significant interactions between AGP supplementation and LPS administration on CAT and HMOX1 transcript levels were evident at both 6 and 24 h.

### Experiment 2

#### Growth Performance and Lymphoid Organ Weights

*E. maxima*/*C. perfringens* co-infected chickens fed diets containing virginiamycin or bacitracin methylene disalicylate had increased body weights at days 22 to 28 post-hatch (days 2–8 following *C. perfringens* infection), compared with co-infected chickens fed the unsupplemented diet (Table 4). In fact, during this time period, body weights of co-infected chickens fed either antibiotic were equal to those of unsupplemented/uninfected chickens. Co-infected chickens fed the virginiamycin-supplemented diet also had increased body weights at days 19–21 post-hatch, compared with unsupplemented/co-infected controls. No differences in normalized weights of the bursa of Fabricus or spleen were seen in any of the four treatment groups (unsupplemented/uninfected, unsupplemented/co-infected, virginiamycin-supplemented/co-infected, bacitracin-supplemented/co-infected).

**Table 4:** Body weights of chickens fed an unsupplemented diet or a diet supplemented with virginiamycin or bacitracin methylene disalicylate with or without *E. maxima*/*C. perfringens* co-infection^a^.

	**Body weight (g/chicken)**					
**Days post-hatch**	**Basal diet**	**Basal diet + Co-infection**	**Virginiamycin + Co-infection**	**BMD + Co-infection**	**Pooled SEM**	**Pooled *P*-value**
1	53.7	52.5	53.8	53.3	0.60	0.424
2	62.6	61.8	63.0	61.8	0.79	0.628
3	76.5	74.6	77.2	74.4	1.17	0.252
4	90.9	90.6	94.3	89.5	1.67	0.200
5	112.2	109.3	115.2	108.2	2.31	0.145
6	128.5	128.1	135.3	124.7	2.79	0.058
7	148.5	147.8	156.9	143.8	3.44	0.058
8	174.9	175.5	184.3	168.6	4.56	0.117
9	206.3	207.8	218.2	200.6	5.63	0.171
10	238.6	241.0	251.2	229.2	6.67	0.145
11	275.7	278.3	291.8	264.9	8.08	0.137
12	310.2	315.1	328.9	304.2	9.46	0.300
13	351.8	354.5	370.9	355.0	10.66	0.569
14	402.3	400.2	426.8	385.7	11.59	0.098
15	439.2	439.0	473.8	428.0	13.59	0.114
16	489.7	491.2	533.6	496.1	13.95	0.092
17	542.1	541.5	599.0	571.1	17.50	0.064
18	610.8	601.9	661.6	639.3	18.67	0.093
19	669.6^a^	669.9^a^	746.2^b^	711.4^a^^b^	21.24	0.031
20	722.4^a^	722.8^a^	807.0^b^	775.6^a^^b^	22.59	0.019
21	789.5^a^^b^	749.0^a^	842.5^b^	817.6^a^^b^	24.01	0.041
22	855.0^a^	734.1^b^	837.1^a^	812.4^a^	26.40	0.008
23	903.0^a^	730.7^b^	859.5^a^	860.9^a^	30.31	0.001
24	965.7^a^	783.9^b^	913.1^a^	920.2^a^	32.87	0.001
25	1068^a^	875.3^b^	1012^a^	1013^a^	34.54	0.001
26	1154^a^	939.8^b^	1097^a^	1098^a^	35.44	0.0003
27	1256^a^	1011^b^	1165^a^	1172^a^	39.34	0.0004
28	1311^a^	1117^b^	1263^a^	1271^a^	37.01	0.002

a *In each row, values with different superscript letters are significantly different (P < 0.05)*.

#### Intestinal Morphometry and Pathology

**Figure 4:**
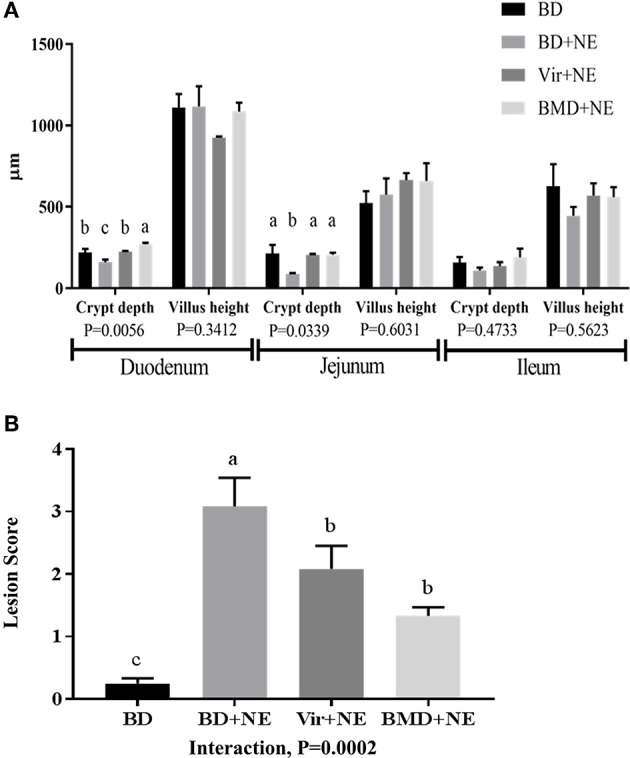
Intestinal morphometry and pathology with or without *NE* challenge. Chickens were fed from hatch with an unsupplemented basal diet (BD) or a diet supplemented with 20 g/ton of virginiamycin (Vir) or 50 g/ton of bacitracin methylene disalicylate (BMD). At 16 days post-hatch, the chickens were infected by oral gavage with 1.0 × 10^4^ oocysts/bird of *E. maxima* or PBS, and at 20 days post-hatch the chickens were infected by oral gavage with 1.0 × 10^9^ colony forming units/bird of *C. perfringens* or PBS, as an experimental model of necrotic enteritis (NE). At 22 days post-hatch, **(A)** intestinal crypt depth and villus height in the duodenum, jejunum, and ileum and **(B)** lesion scores in the jejunum were measured. Vertical bars represent mean ± SEM values (*n* = 4). Bars not sharing the same letters are significantly different at *P* < 0.05.

In the duodenum and jejunum, intestinal crypt depths were increased in *E. maxima*/*C. perfringens*-co-infected chickens fed AGP-supplemented diets at day 22 post-hatch (day 2 following *C. perfringens* infection), compared with unsupplemented/co-infected chickens (Figure 4A). Of note, crypt depths in the jejunum of chickens given either antibiotic, and in the duodenum of chickens given virginiamycin, were equal to those of unsupplemented/uninfected controls, and greater than controls in chickens fed the bacitracin-supplemented diet, at this time. Finally, chickens in the AGP-supplemented/co-infected group exhibited decreased intestinal lesion scores (jejunum) at day 22 post-hatch, compared with unsupplemented/co-infected controls (Figure 4B), despite the fact that there were no differences in crypt depth and villus height in this region.

#### Proinflammatory Cytokine and Antioxidant Enzyme Transcript Levels

**Figure 5:**
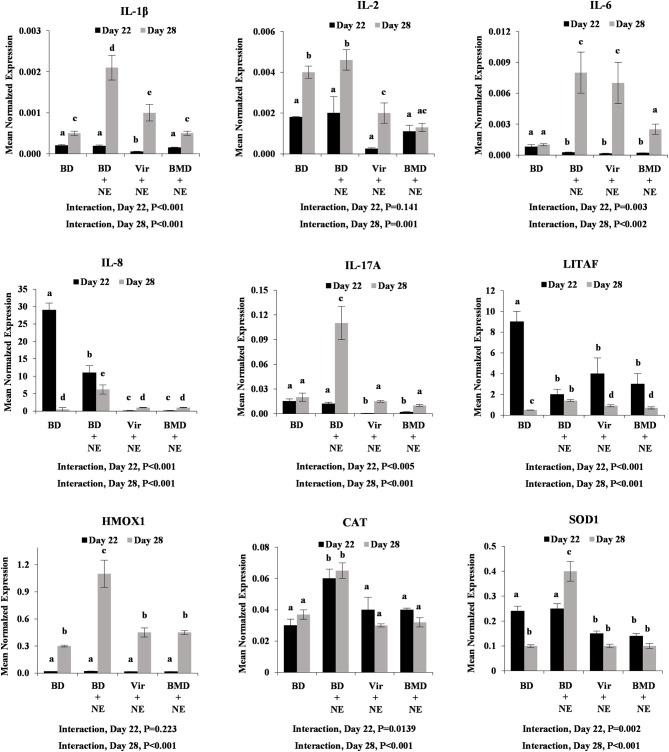
Transcript levels of proinflammatory cytokine and antioxidant enzyme at day 22 and day 28 post-hatch in intestine with or without *NE* challenge. Chickens were fed an unsupplemented basal diet (BD) or a diet supplemented with virginiamycin (Vir) or bacitracin methylene disalicylate (BMD), co-infected with *E. maxima*/*C. perfringens* as an experimental model of necrotic enteritis (NE) or PBS, and the levels of intestinal jejunum transcripts encoding IL-1β, IL-2, IL-6, IL-8, IL-17A, LITAF, HMOX1, CAT, and SOD1 were measured by qRT-PCR and normalized to GAPDH transcripts. Vertical bars represent mean ± SEM normalized transcript levels (*n* = 4). *P*-values for the interaction between AGP supplementation and *E. maxima*/*C. perfringens* co-infection are indicated below each panel. Different letters means significant differences between data.

**Figure 6:**
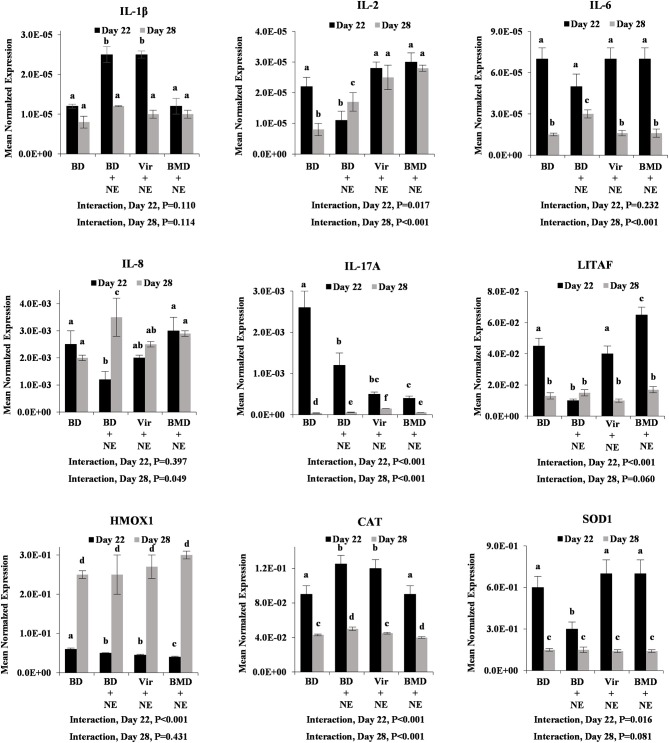
Transcript levels of proinflammatory cytokine and antioxidant enzyme at day 22 and day 28 post-hatch in liver with or without NE challenge. Chickens were fed an unsupplemented basal diet (BD) or a diet supplemented with virginiamycin (Vir) or bacitracin methylene disalicylate (BMD), co-infected with *E. maxima*/*C. perfringens* as an experimental model of necrotic enteritis (NE) or PBS, and the levels of liver transcripts encoding IL-1β, IL-2, IL-6, IL-8, IL-17A, LITAF, HMOX1, CAT, and SOD1 were measured by qRT-PCR and normalized to GAPDH transcripts. Vertical bars represent mean ± SEM normalized transcript levels (*n* = 4). *P*-values for the interaction between AGP supplementation and *E. maxima*/*C. perfringens* co-infection are indicated below each panel. Different letters means significant differences between data.

*E. maxima*/*C. perfringens*-co-infected chickens fed the unsupplemented basal diet had increased levels of intestinal transcripts encoding IL-1β, IL-6, IL-8, IL-17A, HMOX1, CAT, and SOD1 at day 28 post-hatch, compared with unsupplemented and uninfected chickens (Figure 5). Similar to LPS challenge (Figure 3), however, chickens fed either virginiamycin or bacitracin methylene disalicylate and co-infected with *E. maxima* and *C. perfringens* had decreased levels of intestinal cytokine and antioxidant transcripts, compared with unsupplemented and co-infected chickens. In many cases (e.g., IL-8, IL-17A, HMOX1, CAT, SOD1), these transcript were decreased to the levels observed in uninfected birds. At day 22 post-hatch, only gut CAT transcript levels were increased in unsupplemented/co-infected chickens, compared with unsupplemented/uninfected animals, and decreased in AGP-supplemented/co-infected chickens, compared with unsupplemented/ co-infected birds (Figure 5). Significant interactions between AGP supplementation and *E. maxima*/*C. perfringens* co-infection on altered intestinal transcript levels were observed for all transcripts at day 28 post-hatch. This pattern of cytokine and antioxidant enzyme gene expression was less evident in the liver where only IL-1β and CAT at day 22, and IL-8 at day 28, were increased in unsupplemented/co-infected chickens, compared with unsupplemented/uninfected birds, and decreased in AGP-supplemented/co-infected chickens, compared with unsupplemented/ co-infected animals (Figure 6). Significant interactions between AGP supplementation and *E. maxima*/*C. perfringens* co-infection on altered liver transcript levels were observed for all transcripts except IL-1β and IL-6 at day 22 post-hatch, and for IL-2, IL-6, IL-8, IL-17A, and CAT at day 28.

## Discussion

The current study was conducted to elucidate the potential mechanism through which the AGPs, virginiamycin and bacitracin methylene disalicylate, might increase poultry growth performance under conditions simulating exposure to infectious pathogens. As anticipated, increased intestinal levels of inflammatory cytokine transcripts were observed in chickens fed a basal diet and experimentally challenged with *E. coli* LPS, or infected with *E. maxima* and *C. perfringens* as a model of avian necrotic enteritis, compared with unchallenged or uninfected chickens. In contrast, chickens fed an AGP-supplemented diet and challenged with LPS or infected with *E. maxima* and *C. perfringens* had decreased inflammatory cytokine transcript levels, compared with LPS-challenged or pathogen-infected birds fed the unsupplemented diet. More specifically, IL-8 and IL-17A transcripts in AGP-supplemented and LPS-challenged birds, and IL-2, IL-8, and IL-17A transcripts in AGP-supplemented and pathogen-infected birds, were reduced to the levels observed in unsupplemented and unchallenged/uninfected controls. A similar pattern in antioxidant enzyme transcript levels was observed in the intestine of AGP-supplemented, *E. maxima*/*C. perfringens*-infected chickens, but generally not for cytokine or antioxidant enzyme transcripts in the liver following pathogen infection or for intestinal antioxidant enzyme transcripts following LPS challenge. Therefore, the effect of AGP supplementation appeared to be relatively specific for down-regulation of inflammatory cytokine gene expression in the intestine following LPS challenge or live pathogen infection. Interestingly, some differences in the cytokine response between virginiamycin and bacitracin methylene disalicylate were noted, including intestinal IL-2 and IL-8 levels at 24 h following LPS exposure, and intestinal IL-6 levels at day 28 and liver IL1-β and LITAF levels at day 22 following *E. maxima*/*C. perfringens* co-infection. A limitation of the current study is that an anti-inflammatory effect imparted by AGPs in the context of pathogen infection might be attributed to an indirect bacterial effect due absent or low activation of inflammation. An additional drawback to the current study concerns the ability of inflammatory cytokines to generate an anorexic effect that may impact body weight gain in chickens fed an AGP-supplemented diet (10).

Scientific evidence has emerged linking in-feed antibiotic usage during food animal production with the emergence of microbial antibiotic resistance and the potential for this resistance to be transferred to the human population (4). These concerns have led to governmental regulations on the use of AGPs in veterinary medicine, necessitating a timely need for development of antibiotic alternatives that maintain the growth promoting effect while simultaneously supporting optimal animal health. However, limited information is available on how AGPs work, hindering the rational development of antibiotic alternatives (7). An early theory on the mechanism of action of AGPs proposed an altered commensal microflora in the gut of AGP-fed animals, with changes in both microbial quantity and quality and leading to reduced competition for essential nutrients between the host and intestinal microbiome (26, 27). Subsequent studies have supported this antimicrobial hypothesis (8, 12–15, 27–35). As summarized by Niewold (10), however, several lines of evidence are incompatible with the antimicrobial hypothesis: (1) AGPs are effective at doses lower than their minimum inhibitory concentration. (2) AGPs have similar effects on growth for different food production animals despite differences in their intestinal microflora. (3) The growth promoting activity of some antibiotics does not correlate with their antimicrobial activity. (4) Subtherapeutic AGP concentrations promote antibiotic resistance rather than bacterial killing. (5) Non-antibiotic compounds that alter the gut microbiome similar to AGPs do not consistently exhibit growth promoting effects. It is also apparent, however, that subtherapeutic levels of antibiotics can influence the replication, production of virulence factors, and/or susceptibility to host defenses of gut bacteria, which may also contribute to their growth-promoting activity (11), and that cytokines released from inflammatory host cells may induce anorxia (10).

As an alternative to the antimicrobial hypothesis, an anti-inflammatory effect of AGPs has been proposed to account for their growth enhancing activity (10). Twenty years ago, Thomke and Elwinger (36) hypothesized that inflammatory cytokines elicited in response to an immunologic stimulus increase the release of catabolic hormones that reduce muscle mass. The anti-inflammatory hypothesis of AGP action is based on the observations that nutritional support is necessary for generation of a robust inflammatory response, and inflammatory mediators, such as cytokines and chemokines, not only diminish appetite, but also increase catabolism, both of which contribute to reduced weight gain (37). Decreased amino acid requirements have been reported for growing chicks undergoing immunologic stress following challenge with *E. coli* LPS or heat-killed *Staphylococcus aureus*, compared with unchallenged controls (38). Chickens challenged with LPS had reduced body weight gain, which was attributed to increased inflammation in response to the challenge as well as decreased feed intake (39). Chickens undergoing inflammatory stress following injection of Sephadex beads had decreased weight gain and reduced feed intake compared with unchallenged controls, which was reversed by injection of IL-1β (40). In these same studies, *ex vivo* incubation of chicken muscle with exogenous IL-1β increased protein degradation by about 24% compared with controls.

In light of the studies demonstrating that inflammation contributes to decreased chicken weight gain and feed intake, and increased catabolism, what is the evidence that AGPs exhibit anti-inflammatory properties that might be responsible for their growth promoting effect? In this study, AGP dietary supplementation was associated with decreased intestinal IL-8 and IL-17A transcript levels in LPS-challenged chickens, and diminished gut IL-2, IL-8, and IL-17A transcript levels in *E. maxima*/*C. perfringens*-infected birds, compared with unsupplemented and challenged controls. In other studies, chickens fed a bacitracin methylene disalicylate-supplemented diet and infected with *C. perfringens* had decreased levels of transcripts in the intestinal jejunum encoding IL-2 and the proinflammatory cytokine, interferon-γ (IFN-γ), compared with unsupplemented and bacteria-challenged controls (41). In separate studies, chickens fed a bacitracin-supplemented diet and challenged with LPS had decreased intestinal levels of transcripts for IL-8 and the inflammatory cytokine, TNFSF-15, compared with unsupplemented and LPS-challenged controls (26). Chickens fed a diet supplemented with the AGP, salinomycin, and exposed to used poultry production litter contaminated with *Eimeria* and *Clostridium* had decreased levels of intestinal transcript for IFN-γ and TNFSF-15, and increased levels of transcripts for the anti-inflammatory cytokine, IL-10, compared with unsupplemented and pathogen-exposed controls (42). In unchallenged pigs, feed supplementation with subtherapeutic levels of AGPs was associated with decreased levels of a variety of inflammation-related serum proteins, compared with unsupplemented controls (17, 20). Finally, in human studies, administration of erythromycin and related antibiotics, which have been used as AGPs for food animal production, was associated with decreased synthesis of IL-1β, IL-6, IL-8, and TNFα (16). Nevertheless, it is important to mention that other studies have documented increased proinflammatory cytokine expression following AGP administration, which might be related to differences in experimental design, tissue sampling times, and/or age of the experimental animals (24, 26). Taken together with previous reports, the current study suggests that dietary AGPs might increase poultry growth, in part, through down-regulation of pathogen-induced, cytokine-mediated inflammatory responses. Current studies are in progress to characterize the cytokine response of chickens fed with AGPs, including those mediating anti-inflammatory effects (e.g., IL-10). Further microbiome studies are required to clarify the mechanisms through which pro-inflammatory cytokine and tight junction expression might be regulated by AGPs.

## Data Availability Statement

The raw data supporting the conclusions of this manuscript will be made available by the authors, without undue reservation, to any qualified researcher.

## Ethics Statement

The animal study was reviewed and approved by Beltsville Agricultural Research Center Institutional Animal Care and Use Committee.

## Author Contributions

SO and HL conceived and designed the experiments. SO and YL performed animal experiments and data analysis. DB performed statistical analysis. SO and EL wrote the paper. All authors offered a critical review of the manuscript. All authors offered a critical review of the manuscript, read, and approved the final manuscript.

### Conflict of Interest

DB was employed by Pancosma and is currently employed at Land O'Lakes. The remaining authors declare that the research was conducted in the absence of any commercial or financial relationships that could be construed as a potential conflict of interest.
